# Determinants of High Salt Intake in Croatian Adults: Evidence from 24-Hour Urinary Sodium Excretion in the EH-UH 2 Study

**DOI:** 10.3390/life16071201

**Published:** 2026-07-20

**Authors:** Bojan Jelaković, Mihaela Marinović Glavić, Ana Stupin, Marija Domislović, Ana Jelaković, Lovorka Bilajac, Andrej Belančić, Matea Bilobrk, Marta Bolješić Dumančić, Mirjana Fuček, Lana Gellineo, Andrea Gross Bošković, Josipa Josipović, Verica Kralj, Sanja Kolarić Kravar, Ivan Pećin, Vladimir Prelević, Danilo Radunović, Petar Šušnjara, Vanja Vasiljev, Marijana Živko, Donatella Verbanac, Željko Reiner

**Affiliations:** 1 University Hospital Center Zagreb, 10000 Zagreb, Croatia; domislovic.marija@gmail.com (M.D.); anajelakovic9@gmail.com (A.J.); mirjana.fucek@kbc-zagreb.hr (M.F.); lana.gellineo@gmail.com (L.G.); ivanpecin@yahoo.com (I.P.); marijana.zivko3@gmail.com (M.Ž.); zeljko.reiner@kbc-zagreb.hr (Ž.R.); 2 School of Medicine, University of Zagreb, 10000 Zagreb, Croatia; 3 Croatian Hypertension League, 10000 Zagreb, Croatia; anacavka@mefos.hr (A.S.); lovorka.bilajac@uniri.hr (L.B.); a.belancic93@gmail.com (A.B.); mateabilobrk5@gmail.com (M.B.); boljesic1997@gmail.com (M.B.D.); vladimir.scopheurope@gmail.com (V.P.); danilo.radunovic2@gmail.com (D.R.); psusnjara@kifos.hr (P.Š.); vanjav@medri.uniri.hr (V.V.); donatella.verbanac@pharma.unizg.hr (D.V.); 4 Faculty of Medicine, University of Rijeka, 51000 Rijeka, Croatia; 5 Faculty of Medicine, Josip Juraj Strossmayer University of Osijek, 31000 Osijek, Croatia; 6 Teaching Institute of Public Health of Primorje—Gorski Kotar County, 51000 Rijeka, Croatia; 7 Croatian Agency for Agriculture and Food, 31000 Osijek, Croatia; andrea.gross-boskovic@hapih.hr; 8 School of Medicine, Catholic University, 10000 Zagreb, Croatia; 9 University Hospital Centre Sestre Milosrdnice, 10000 Zagreb, Croatia; 10 Croatian Institute of Public Health, 10000 Zagreb, Croatia; verica.kralj@hzjz.hr; 11 Ministry of Agriculture, Forestry and Fisheries, Directorate for Livestock and Food Quality, 10000 Zagreb, Croatia; sanja.k-kolaric@mps.hr; 12 Clinical Centre of Montenegro, Clinic for Nephrology, 81000 Podgorica, Montenegro; 13 School of Medicine, University of Montenegro, 81000 Podgorica, Montenegro; 14 Faculty of Kinesiology Osijek, Josip Juraj Strossmayer University of Osijek, 31000 Osijek, Croatia; 15 Croatia University Libertas, 10000 Zagreb, Croatia; 16 Faculty of Pharmacy and Biochemistry, University of Zagreb, 10000 Zagreb, Croatia

**Keywords:** epidemiology, lifestyle, predictors, salt, socioeconomic status, 24-h urinary sodium excretion

## Abstract

Although significant progress has been made in reducing salt consumption over the past decade, most Croatian adults still exceed the recommended intake. This study aimed to identify demographic, socioeconomic, lifestyle, and clinical factors associated with high salt intake in the general adult population of Croatia. We included a random sample of 1067 adults with reliable 24-h urine samples. A structured questionnaire was used to collect sociodemographic, lifestyle, and clinical data. Anthropometric and blood pressure (BP) measurements were performed. Laboratory analyses included the measurement of sodium, potassium, and creatinine in adequate 24-h urine samples, as well as relevant cardiometabolic and kidney-related biomarkers. Participants consuming > 10 g/day of salt were older and had higher systolic blood pressure, fasting glucose, serum uric acid, and triglyceride levels, lower HDL cholesterol levels, and greater BMI. High salt intake was more common among men, ex-smokers, participants with diabetes, and those with lower socioeconomic status (SES). In multinomial logistic regression analysis, male sex, diabetes, higher systolic BP, more frequent processed meat consumption, lower fish consumption, residence outside the Adriatic region, and higher ePWV were associated with high salt intake. High salt intake was associated with lower socioeconomic status, residence in rural and continental areas, obesity, former smoking, and less favorable dietary patterns. Conversely, lower salt intake was associated with residence in the Adriatic region and dietary patterns characterized by more frequent consumption of fish and olive oil.

## 1. Introduction

Hypertension (HT) is a major public health issue in Croatia, where the EH-UH 2 national survey (Epidemiology of Hypertension and Salt Intake in Croatia) reported a prevalence of 50.1% in adults in 2020, and Eurostat data ranked Croatia highest among European Union countries for elevated blood pressure in 2019 [1,2]. The most recent Global Burden of Disease report identified high blood pressure as the leading contributor to disability-adjusted life years (DALY) in Croatia [3]. According to official analyses by the Croatian Bureau of Statistics and the Croatian Institute of Public Health, HT is a major contributor to mortality in Croatia, while ischemic heart disease remains the leading cause of death. Together, these findings underscore the substantial public health burden of hypertension in Croatia [4]. Notably, HT is the leading cause of morbidity among women in Croatia, underscoring its demographic impact. High dietary salt intake is a well-established modifiable risk factor for HT. Evidence from international and Croatian studies, including the Croatian 24-h mapping study, demonstrates a strong association between salt consumption and BP values [2,5,6]. Consequently, salt restriction is universally recommended in all major HT guidelines as a first-line lifestyle intervention with the highest level of evidence [7,8,9]. Despite global recommendations from the World Health Organization (WHO) to limit salt intake to less than 5 g per day, only 13.7% of Croatian adults achieved this target [5,10]. Average salt intake in Croatia remains substantially above recommended levels and varies by geographic region and by place of residence, reflecting heterogeneous dietary exposures across the population [5]. Although population-wide salt reduction is considered a highly cost-effective strategy for preventing hypertension and improving cardiovascular, kidney, and metabolic health, Croatia still lacks comprehensive national programs addressing both excessive salt intake and hypertension. Effective salt-reduction policies require a clear understanding of dietary habits, the food environment, socioeconomic conditions, and relevant comorbidities within the target population. This study represents a secondary analysis of the EH-UH 2 dataset, which was previously used to estimate national salt, potassium, and iodine intake in Croatian adults [5]. The aim of this study was to identify demographic, socioeconomic, lifestyle, and clinical factors associated with high salt intake, assessed by 24-h urinary sodium excretion, in a large nationally representative sample of the non-institutionalized adult Croatian population from the EH-UH 2 study. These findings are intended to support the development of targeted salt reduction strategies in clinical practice and public health.

## 2. Materials and Methods

### 2.1. Study Design

This cross-sectional study was conducted as part of the EH-UH 2 study (Epidemiology of Hypertension and Salt Intake in Croatia), a nationwide survey based on a randomized sample of non-institutionalized Croatian adults. The study collected data on anthropometric measurements, demographic and lifestyle characteristics, and biological markers to assess the prevalence of HT and associated CKM risk factors. The detailed sampling procedure and the core EH-UH 2 protocol have been reported previously [2,11]; therefore, only methods directly relevant to the present analysis are summarized here. The flow diagram of the final sample is provided in the Supplementary Materials (Figure S1). Inclusion criteria were age ≥18 years and signed informed consent. Exclusion criteria were terminal illness, dementia, paresis, limb amputation or immobilization, acute illness, COVID-19 infection within the previous three months, pregnancy or lactation, and the absence of signed informed consent. This manuscript reports a secondary analysis of the EH-UH 2 dataset, focusing specifically on factors associated with high salt intake. Detailed information on participant recruitment and response rates was reported previously [5]. The overall EH-UH 2 survey response rate was 73%. The estimated completion rate for the 24-h urine collection was 94.5%, while 84.1% of the collected samples met the predefined adequacy criteria, resulting in an estimated overall rate of adequate 24-h urine collection of approximately 61%. Compared with the general Croatian adult population, participants included in the EH-UH 2 sample were older and included a lower proportion of men, reflecting a higher response rate among older individuals and women [5].

### 2.2. 24-Hour Urine Collection and Laboratory Procedures

Participants received 2.5-L plastic containers and standardized verbal and written instructions during a home visit. They were instructed to discard the first morning void, collect all urine over the subsequent 24 h, and end the collection with the first void of the following morning. At the outpatient visit, total urine volume was measured, samples were homogenized, and three 2-mL aliquots were stored at 4 °C and transported on the same day to the Central Laboratory of the Clinical Hospital Centre Zagreb. Quality assurance procedures were applied to minimize inclusion of incomplete urine collections. A 24-h urine sample was considered incomplete if: (1) the start or end time was not recorded; (2) collection duration was outside the acceptable range of 22–26 h; (3) total urine volume was <500 mL; (4) the participant was menstruating; or (5) the participant reported missing more than a few drops of urine during collection. Additional predefined criteria indicating a high probability of incomplete collection were urinary creatinine values outside two standard deviations of the sex-specific distributions (5.9–26.0 mmol/24 h for men and 4.0–16.4 mmol/24 h for women).

### 2.3. Study Sample and Salt Intake Classification

Only participants who adhered to the study protocol and met quality control criteria for complete 24-h urine collection were included in the analysis. The final study sample comprised 1067 individuals aged 18–89 years. Of these, 956 participants had complete demographic, clinical, and laboratory data available for the present analyses. Based on estimated daily salt intake derived from 24-h urinary sodium excretion, participants were categorized into three groups: <5 g/day (*n* = 131; 13.7%), 5–10 g/day (*n* = 465; 48.6%), and >10 g/day (*n* = 360; 37.6%). The survey followed the guidelines set by the Declaration of Helsinki and Good Clinical Practice [12]. The Ethics Committee of the School of Medicine, University of Zagreb, granted ethical approval for the survey (RN 380-59-10106-17-100/207) on 13 July 2017.

### 2.4. Statistical Analysis

Data were entered into MS Excel (Microsoft Office LTSC Professional Plus 2021) and processed in IBM SPSS Statistics 28.0.0.0. (IBM Corporation, Armonk, NY, USA). The normality of continuous variables was assessed using the Kolmogorov–Smirnov test. Descriptive statistics included measures of central tendency: mean and standard deviation for normally distributed variables, and median with interquartile range (IQR) for non-normally distributed variables. Categorical variables were expressed as absolute numbers and percentages (*n*, %). We used Student’s *t*-test for normally distributed variables, and Mann–Whitney U-test for non-normally distributed variables. The Kruskal–Wallis test, a non-parametric statistical method, was employed to compare three or more independent groups of data measured on an ordinal or interval scale. Correlations were assessed using Pearson’s correlation coefficient for normally distributed variables and Spearman’s rank correlation coefficient for non-normally distributed variables. Stepwise backward regression was used to identify the most relevant predictor variables from regression models. Multinomial logistic regression was used to examine factors associated with membership in the three salt-intake categories, using the 5–10 g/day category as the reference outcome. Odds ratios (ORs) with 95% confidence intervals were calculated to assess the strength of association between potential predictors and high salt intake. Participants were compared across three salt-intake categories (<5, 5–10, and >10 g/day). Socioeconomic and food-frequency variables were entered as ordinal predictors, with higher scores indicating better SES and more frequent consumption. Socioeconomic, lifestyle, and dietary variables were derived from the questionnaire items as follows: (a) Average monthly household income (HRK): <2500 = 1; 2500–3499 = 2; 3500–4999 = 3; 5000–10,000 = 4; >10,000 = 5. (b) Years of formal education: 4–8 years = 1; 8–12 years = 2; >12 years = 3. (c) Highest educational qualification: unskilled worker (NKV) = 1; highly skilled worker (VKV) = 2; secondary education (SSS) = 3; post-secondary or associate degree (VŠS) = 4; university degree (VSS) = 5. (d) Frequency of processed meat, fish, and poultry consumption: never = 1; very rarely (a few times per month) = 2; up to twice per week = 3; and every day or almost every day = 4. The type of fat used most frequently was coded as follows: vegetable oil = 1, pork lard = 2, olive oil = 3, and margarine = 4. As the primary objective of the present study was to identify factors associated with high salt intake, only the contrast between >10 g/day and 5–10 g/day is presented and interpreted in the main analysis. Reference categories and coding directions are provided in the regression tables. When the original coding produced Exp(B) values below 1.00, reciprocal odds ratios were calculated to present the associations consistently in the direction of higher odds of high salt intake. For all tests, *p* < 0.05 was deemed statistically significant.

## 3. Results

### 3.1. Demographic, Clinical and Laboratory Data of Participants Divided into Three Salt Intake Subgroups

Table 1 presents the demographic and clinical characteristics of the participants, whereas Table 2 presents their laboratory findings. Participants consuming more than 10 g/day were significantly older than those consuming less than 5 g/day, whereas no significant difference was observed between the 5–10 g/day and >10 g/day groups (*p* = 0.03). Both systolic and diastolic BP increased progressively across the salt-intake categories and were highest in the >10 g/day group (*p* < 0.001). Height, weight, BMI, waist circumference, and body surface area all increased across salt intake groups and were significantly higher in group 3 compared with groups 1 and 2 (*p* < 0.001). Estimated pulse wave velocity was highest in the >10 g/day group and differed significantly from that observed in the <5 g/day group. Higher salt intake was associated with several adverse metabolic and kidney markers. Participants consuming >10 g/day had higher fasting glucose and serum uric acid levels, higher triglycerides, and lower HDL cholesterol compared with lower intake groups. Estimated glomerular filtration rate (eGFR) was modestly higher in the high-intake group; however, given the cross-sectional design, this finding should be interpreted as an association and not as evidence of a causal mechanism. Urinary potassium excretion increased with salt intake, and the urinary sodium-to-potassium ratio worsened markedly in those with high intake. Cardiac biomarkers showed no significant differences.

### 3.2. Proportion of Participants with Recommended Salt Intake and High Salt Intake

A significantly higher proportion of men than women consumed more than 10 g of salt per day (Table 3). Ex-smokers had the highest prevalence of high salt intake, followed by never-smokers and current smokers. The prevalence of high salt intake increased progressively across BMI and waist-circumference categories. Participants in the highest education and household-income categories had the lowest prevalence of high salt intake. High salt intake was also more common among participants with diabetes or a history of myocardial infarction than among those without these conditions. Normotensive participants had the lowest prevalence of high salt intake, whereas participants with untreated hypertension had the lowest prevalence of recommended salt intake. High salt intake was most common among participants who consumed processed meat most frequently and was less common among those with more frequent fish consumption. It was also more prevalent among participants living in rural areas and in continental Croatia than among those living in urban areas and the Adriatic region.

### 3.3. Factors Associated with High Salt Intake

In the stepwise backward linear regression analysis, several clinical, anthropometric, socioeconomic, dietary, and regional variables remained in the final model. Higher systolic blood pressure, greater body surface area, more years of education, more frequent processed meat consumption, and residence in the continental region were positively associated with daily salt intake. The coefficients and confidence intervals for all variables retained in the model are presented in Table 4. When all variables were included in the multinomial logistic regression model (R^2^ = 0.225), statistically significant factors associated with higher odds of high salt intake (>10 g/day) were male sex (OR = 3.36), non-smoking status (OR = 1.48), diabetes (OR = 2.69), higher systolic BP (OR = 1.04), more frequent processed meat consumption (OR = 1.44), lower fish consumption (OR = 1.78), residence in the continental region compared with the Adriatic region (OR = 1.55), and higher ePWV (OR = 1.62) (Table 5).

## 4. Discussion

Our study found that residence in the Adriatic region, where fish and olive oil are commonly consumed, was associated with lower salt intake. This likely reflects the persistence of Mediterranean dietary patterns in the Adriatic region. The analyses identified male sex, non-smoking status, diabetes, higher systolic BP, more frequent processed meat consumption, lower fish consumption, residence in the continental region, and higher ePWV as factors associated with high salt intake. However, the multinomial model indicated modest explanatory ability. This is not unexpected, as salt intake is influenced by numerous dietary, behavioral, environmental, and individual factors that were not fully captured in the present analysis. Therefore, our findings should be interpreted as identifying characteristics associated with high salt intake rather than as providing a comprehensive predictive model. Higher ePWV was observed among participants with high salt intake. However, because ePWV is calculated from age and BP, and because of the cross-sectional design of the study, this association should be interpreted as exploratory. In our study, high salt intake was particularly evident among non-smokers, with the highest values observed among ex-smokers. Ex-smokers had higher BMI and a greater prevalence of obesity. Therefore, their higher salt intake may reflect greater overall food intake rather than smoking status itself. The observed high salt intake among participants with diabetes may indicate insufficient dietary awareness, suboptimal adherence to dietary recommendations, or inadequate implementation of nutritional counseling in routine care.

### 4.1. Sociodemographic Characteristics and Salt Intake

Younger participants and women had lower salt intake, which is consistent with previous studies [13,14,15,16,17,18,19,20]. Age-related declines in salt taste sensitivity may contribute to a greater preference for salty foods among older individuals [13,14,15,16]. Men consumed more salt than women, possibly because of their generally higher energy intake and potential sex-related differences in taste perception [16]. Some studies have reported that salt intake decreases with age in men but increases in women, whereas others have found lower sodium excretion in middle-aged women than in younger women [21,22,23]. Although previous studies have reported inconsistent sex-specific age trends, participants in the higher salt-intake categories in our study tended to be older. In our study, lower socioeconomic status, educational attainment, and household income were associated with high salt intake. These findings are consistent with previous evidence showing that dietary risk factors often cluster with socioeconomic disadvantages. Participants with lower levels of education and occupational qualification had higher salt intake. Similar gradients have been reported in the UK, Finland, Italy, and Montenegro [24,25,26,27]. Socioeconomic indicators were included as covariates and remained relevant in characterizing individuals with high salt intake, but the present analysis primarily focused on high salt intake. Participants living in rural areas had higher salt intake, lower potassium intake, and an unfavorable sodium-to-potassium ratio, indicating poorer dietary quality. Regionally, the lowest odds of consuming more than 10 g/day of salt were observed in the Adriatic region, supporting the influence of regional dietary patterns [27,28].

### 4.2. Blood Pressure, Hypertension, and Heart Rate

We observed a positive association between salt intake and BP, consistent with well-established evidence [22,29,30,31,32,33,34,35]. A Portuguese study reported higher daily salt intake among participants with hypertension than among normotensive participants [36]. In the CARDIA study, an average daily salt intake of 14 g was associated with a 53% higher risk of developing HT compared to an intake of about 3 g per day, over 25 years of follow-up [37,38]. Hypertensive participants consumed significantly more salt than normotensive individuals. Normotensive participants had the highest proportion of recommended salt intake, whereas participants with HT more frequently consumed >10 g/day. A few large-scale studies have explored the link between salt intake and heart rate [39,40]. A meta-analysis found that a reduction in dietary salt intake significantly increased heart rate [41]. The Swiss Salt Study reported that participants with a daily salt intake higher than recommended had a significantly lower heart rate [42]. This is in line with our results showing that participants who consumed more than 10 g of salt per day had a significantly lower heart rate. The impact of salt reduction on heart rate requires further investigation.

### 4.3. Obesity and Metabolic Risk

High salt consumption has been associated with several metabolic risk factors. Each additional gram of daily salt intake was linked to a 26% higher risk of obesity [20]. In our study, salt intake was positively associated with BMI, waist circumference, and body surface area. Participants with normal waist circumference and lower body surface area consumed significantly less salt than those with higher values. Body surface area and waist circumference were stronger predictors of salt intake than BMI, suggesting that visceral adiposity may be more relevant than overall obesity.

In the INTERMAP study, salt intake was positively associated with BMI and with the prevalence of overweight and obesity, which is consistent with our results [43]. Participants with normal waist circumference and lower body surface area consumed significantly less salt than those with higher waist circumference and higher body surface area, respectively. The African-PREDICT study found that body surface area was the only independent predictor of high salt intake [44]. In the multivariable analysis, diabetes was significantly associated with higher salt intake, consistent with findings from previous studies [45,46]. In our cohort, participants with a salt intake of >10 g/day had higher triglyceride and lower HDL-cholesterol values compared to the group with the recommended salt intake. These results are consistent with the Mendelian randomization analysis in which increased sodium excretion was associated with lower HDL-cholesterol and higher triglyceride levels [47]. Li et al. showed that participants with low HDL had higher 24-h urinary sodium excretion [48,49]. Conversely, the DASH study did not find any significant association between reduced salt intake and alterations in lipid parameters [50].

### 4.4. Arterial Stiffness, Cardiac Biomarkers and Cardiovascular Events

The association between ePWV and salt intake has not been extensively investigated in population-based settings, and current evidence on this relationship remains limited. In our study, higher salt intake was associated with higher ePWV. However, because ePWV is derived from age and BP, and because of the cross-sectional design of the study, this finding should be interpreted cautiously. Rather than implying a direct causal effect of salt intake on arterial stiffness, our results suggest that high salt intake may cluster with an unfavorable vascular risk profile.

Our findings are consistent with previous studies reporting an association between higher salt intake and increased arterial stiffness [51,52,53,54]. In our study, the lowest prevalence of myocardial infarction was observed among participants consuming less than 5 g/day of salt. This is in line with other studies which reported that high salt intake was associated with higher risk of coronary heart disease and CV death [55,56]. Many studies explored salt intake and heart failure [57,58,59]. The Heart Failure Adherence and Retention trial reported higher risk of death and hospitalization for heart failure among participants randomized to very restricted salt intake compared with those randomized to unrestricted intake [60]. However, sub analyses indicated that very restricted salt intake was associated with higher risk only in patients not receiving renin-angiotensin blockers. There is inconsistency in reports and paucity of robust high-quality evidence about the effect of salt restriction on outcomes in patients with heart failure [61,62]. NT-proBNP showed a significant correlation with salt intake and was retained in the stepwise backward linear regression model. These results are concordant with other reports [63,64]. Joosten et al. suggested that NT-proBNP may help identify individuals who are more vulnerable to the harmful CV effects of high sodium intake, and in the DASH study, NT-proBNP decreased in the low-sodium arm compared to the high-sodium arm [65,66]. Previous studies have reported an association between high sodium intake and an increased risk of stroke and stroke-related mortality [48,67,68].

### 4.5. Kidney Function and Albuminuria

In our cohort, participants consuming >10 g/day of salt had significantly higher eGFR than those consuming <5 g/day. In the regression analysis, each 1 mL/min/1.73 m^2^ increase in eGFR was associated with 1.2% higher odds of consuming >10 g/day of salt. This finding is consistent with previous studies suggesting that high sodium intake may be associated with glomerular hyperfiltration. Krikken et al. reported that high sodium intake increased the filtration fraction in participants with obesity, but not in lean participants [69], while Mallamaci et al. found that salt intake was associated with increased GFR independently of salt-sensitivity status and the renin–aldosterone system [70]. Longitudinal observational studies have reported that higher sodium intake may be associated with a faster decline in eGFR and a higher risk of CKD [71,72]. These findings provide a possible context for our cross-sectional observation but do not establish causality in the present cohort. We also observed a positive association between salt intake and the albumin-to-creatinine ratio. This is consistent with findings from a study of patients with HT, in which albuminuria was significantly higher in the high-salt-intake group and urinary sodium excretion was an independent predictor of albuminuria [73]. In our study, however, salt intake did not differ significantly between participants with CKD and those with normal kidney function. Because dietary counseling, adherence to salt-reduction recommendations, and barriers to dietary change were not assessed, the reasons for this finding cannot be determined from the present data. Previous studies have identified several barriers to adherence to low-salt diets among patients with CKD, including concerns regarding food palatability [74,75,76]; however, these factors were not evaluated in our cohort.

### 4.6. Dietary Patterns and Smoking

In our population, the lowest odds of consuming more than 10 g/day of salt were associated with living in the Adriatic region of Croatia compared to the continental area. Geographical variations in salt consumption were recorded across Europe. In Italy, sodium intake showed a clear North–South gradient, like our observations in Croatia [26]. In the UK, Scotland had the highest intake due to a diet rich in processed and salty foods [24]. More frequent processed-meat consumption was associated with a lower prevalence of recommended salt intake and a higher prevalence of intake exceeding 10 g/day. Each higher category of processed meat consumption increased daily salt intake by 0.7 g. The results presented are consistent with other studies [77,78,79,80]. Importantly, we also found that more frequent fish consumption was associated with the recommended daily salt intake. Previous studies have reported a lower prevalence of hypertension among individuals who consume fish more frequently, possibly reflecting lower sodium and higher potassium intake [81,82]. Interestingly, we found that each increase in the frequency of olive oil consumption decreased daily salt intake by 0.5 g. All these findings indicate that the Adriatic diet and Mediterranean lifestyle are associated with lower salt intake. This finding is consistent with previous studies reporting differences between continental and Adriatic regions of Croatia [83,84]. It would also be valuable to compare our findings with data from other Mediterranean countries.

Chronic smoking impairs taste perception, especially for salty flavors, raising salt taste thresholds [85,86,87]. Large-scale population studies have shown that current smoking is associated with salt-seeking behaviors, indicating a preference for salty foods among smokers [85]. In contrast, a Taiwanese study found no direct association between smoking and salt intake [88]. In our study, the highest proportion of individuals who consumed >10 g/day was observed among ex-smokers. However, ex-smokers had higher BMI and a greater prevalence of obesity. Their higher salt intake may reflect greater overall food consumption rather than an independent effect of former smoking status. Several studies, including the present study, have identified an association between lower socioeconomic status and higher salt intake. In our study, high salt intake was consistently associated with lower SES. We observed that salt intake increased by 0.78 g/day for each lower level of general education, and by 0.53 g/day for each lower level of professional qualification.

### 4.7. Strengths and Limitations of the Study

This study has several limitations. First, the cross-sectional design limits the ability to infer causality and allows only the assessment of associations between salt intake and the analyzed clinical, biochemical, and lifestyle parameters. Therefore, the observed relationships should not be interpreted as causal. In addition, although several relevant covariates were included in the analyses, the possibility of residual confounding or confounding bias cannot be excluded.

Second, salt intake was estimated from a single 24-h urine collection, which may not fully capture day-to-day intraindividual variability in salt consumption and could therefore lead to misclassification of usual intake. However, most large epidemiological studies rely on a single 24-h urine sample because repeated collections are logistically demanding. Third, para-aminobenzoic acid was not used to verify the completeness of urine collection; instead, total urinary creatinine excretion was applied as an accepted alternative criterion for assessing sample adequacy. Fourth, 24-h urinary urea excretion was not measured, which limited the assessment of protein intake and related dietary patterns. Finally, serum and urinary magnesium, calcium, and magnesium-to-creatinine ratios were not measured; therefore, we could not evaluate the potential contribution of broader mineral metabolism to BP, vascular stiffness, kidney function, and cardiometabolic risk. Given the exploratory nature of the analyses and the large number of statistical comparisons, no formal adjustment for multiple testing was applied. Therefore, findings with marginal statistical significance should be interpreted cautiously and considered hypothesis-generating until confirmed in independent studies.

Our study also has several important strengths. Unlike many previous studies, we reported the quality of urine collection and applied rigorous criteria for assessing the adequacy of 24-h urine samples. The study was conducted on a large, nationally representative sample, which makes the results applicable to the general adult population in Croatia. Furthermore, adherence to WHO protocols enables cross-country comparisons.

## 5. Conclusions

Higher salt intake was observed among rural residents, individuals living in continental Croatia, older adults, men, and participants with lower socioeconomic status, obesity, and cardiometabolic or kidney-related comorbidities. High salt intake was associated with more frequent consumption of processed meat, whereas lower salt intake was associated with more frequent fish consumption, use of olive oil, and residence in the Adriatic region, suggesting that the Adriatic diet and Mediterranean lifestyle may contribute to more favorable dietary patterns. Nearly 40% of the Croatian adult population still consumes more than 10 g of salt per day, despite previous efforts to reduce salt intake. These findings highlight the need for continued population-wide salt-reduction initiatives, with particular attention to rural communities and socioeconomically disadvantaged groups. Further reductions in the salt content of bread, bakery products, processed meats, and other commercially processed foods should remain a national public health priority. Finally, the broader promotion of the Adriatic diet and Mediterranean lifestyle may contribute to healthier dietary patterns and lower salt intake in Croatia.

## Figures and Tables

**Table 1:** Demographic and clinical characteristics of participants divided into three salt intake subgroups.

	Whole Group	<5 g/Day	5–10 g/Day	>10 g/Day	*p*
Age (Years)					
Mean (SD)	56.7 (13.8)	53.7 (15.2)	57.1(14.0)	57.4 (13.0)	1–2 0.04 2–3 1.00 1–3 0.03
SE	0.4	1.3	0.6	0.6	
95% CI	55.9–57.6	51.1–56.3	55.8–58.4	56.0–58.7	
Systolic blood pressure (mmHg)					
Mean (SD)	133.3 (19.1)	127.1 (20.9)	131.7 (17.9)	137.7 (18.9)	1–2 0.041 2–3 0.001 1–3 <0.001
SE	0.6	1.8	0.8	1.0	
95% CI	132.1–134.6	123.5–130.7	130.1–133.3	135.8–139.7	
Diastolic blood pressure (mmHg)					
Mean (SD)	82.3 (10.0)	80.3 (11.0)	81.0 (9.3)	84.5 (10.1)	1–2 1.000 2–3 0.001 1–3 <0.001
SE	0.3	0.9	0.4	0.5	
95% CI	81.6–82.9	78.3–82.3	80.2–81.9	83.5–85.6	
Heart rate (beat/min)					
Mean (SD)	76.1 (12.3)	77.6 (13.3)	77.0 (12.8)	74.4 (11.2)	1–2 1.000 2–3 0.012 1–3 0.046
SE	0.4	1.1	0.6	0.6	
95% CI	75.3–76.9	75.2–79.9	75.8–78.2	73.3–75.6	
Height (cm)					
Mean (SD)	169.8 (9.4)	169.1 (9.3)	168.5 (9.1)	171.8 (9.5)	1–2 1.000 2–3 <0.001 1–3 0.018
SE	0.3	0.8	0.4	0.5	
95% CI	169.2–170.4	167.5–170.8	167.6–169.3	170.8–172.8	
Weight (kg)					
Mean (SD)	82.1 (16.8)	77.9 (16.4)	79.0 (15.6)	87.8 (17.1)	1–2 1.000 2–3 <0.001 1–3 0.001
SE	0.5	1.4	0.7	0.9	
95% CI	81.0–83.2	75.1–80.8	77.5–80.4	85.9–89.6	
Body mass index (kg/m^2^)					
Mean (SD)	28.4 (5.1)	27.1 (4.7)	27.8 (4.9)	29.7 (5.1)	1–2 0.546 2–3 <0.001 1–3 <0.001
SE	0.1	0.4	0.2	0.2	
95% CI	28.0–28.7	26.3–27.9	27.3–28.2	29.1–30.2	
Waist circumference (cm)					
Mean (SD)	97.5 (15.4)	92.5 (14.4)	95.4 (15.7)	102.0 (14.6)	1–2 0.183 2–3 <0.001 1–3 <0.001
SE	0.5	1.2	0.7	0.7	
95% CI	96.5–98.5	90.0–95.1	93.9–96.8	100.5–103.6	
Body surface area (m^2^)					
Mean (SD)	1.9 (0.2)	1.9 (0.2)	1.9 (0.2)	2.0 (0.2)	1–2 1.000 2–3 <0.001 1–3 <0.001
SE	0.007	0.0	0.01	0.01	
95% CI	1.9–1.9	1.8–1.9	1.8–1.9	2.0–2.0	
Estimated pulse wave velocity (ePWV, m/s)					
Mean (SD)	9.8 (2.2)	9.2 (2.3)	9.7 (2.2)	10.0 (2.1)	1–2 0.132 2–3 0.144 1–3 0.003
SE	0.07	0.2	0.1	0.1	
95% CI	9.6–9.9	8.8–9.7	9.5–9.9	9.8–10.3	

**Table 2:** Laboratory data of participants divided into three salt intake subgroups.

	Whole Group	<5 g/Day	5–10 g/Day	>10 g/Day	*p*
Fasting blood glucose (mmol/L)					
Mean (SD)	5.18 (1.674)	4.88 (1.229)	5.14 (1.567)	5.34 (1.904)	1–2 0.331 2–3 0.256 1–3 0.021
SE	0.052	0.107	0.069	0.095	
95% CI	5.087–5.292	4.672–5.099	5.011–5.285	5.153–5.529	
Serum creatinine (µmol/L)					
Mean (SD)	73.55 (22.443)	75.14 (33.227)	72.62 (22.001)	74.19 (18.195)	1–2 0.754 2–3 0.897 1–3 1.000
SE	0.698	2.892	0.982	0.911	
95% CI	72.18–74.92	69.42–80.87	70.69–74.55	72.40–75.98	
Estimated glomerular filtration (eGFR, CKD Epi equation) (mL/min/1.73 m^2^)					
Mean (SD)	91.83 (20.875)	88.48 (20.736)	91.19 (20.867)	93.73 (20.797)	1–2 0.561 2–3 0.210 1–3 0.038
SE	0.651	1.818	0.933	1.045	
95% CI	90.553–93.111	84.890–92.086	89.360–93.027	91.681–95.790	
Albumin-to-creatinine ratio (urine mg/g)					
Mean (SD)	20.82 (116.183)	11.39 (16.900)	16.26 (64.168)	29.73 (172.081)	1–2 1.000 2–3 0.307 1–3 0.390
SE	3.813	1.517	3.041	9.082	
95% CI	13.339–28.309	8.394–14.403	10.288–22.245	11.869–47.591	
Serum uric acid (µmol/L)					
Mean (SD)	295.22 (79.689)	288.37 (82.852)	289.50 (77.422)	304.70 (80.718)	1–2 1.000 2–3 0.013 1–3 0.122
SE	2.478	7.211	3.452	4.041	
95% CI	290.36–300.08	274.11–302.64	282.72–296.28	296.75–312.64	
Total cholesterol (mmol/L)					
Mean (SD)	5.36 (1.118)	5.44 (1.260)	5.39 (1.086)	5.30 (1.107)	1–2 1.000 2–3 0.604 1–3 0.601
SE	0.034	0.109	0.048	0.055	
95% CI	5.299–5.436	5.229–5.663	5.303–5.493	5.193–5.411	
Triglycerides (mmol/L)					
Mean (SD)	1.62 (1.032)	1.57 (0.840)	1.52 (0.890)	1.72 (1.232)	1–2 1.000 2–3 0.043 1–3 0.440
SE	0.032	0.073	0.039	0.061	
95% CI	1.557–1.683	1.427–1.717	1.474–1.630	1.601–1.844	
HDL cholesterol (mmol/L)					
Mean (SD)	1.44 (0.371)	1.48 (0.403)	1.49 (0.377)	1.38 (0.341)	1–2 1.000 2–3 <0.001 1–3 0.013
SE	0.011	0.035	0.016	0.017	
95% CI	1.425–1.470	1.417–1.555	1.458–1.524	1.346–1.414	
LDL cholesterol (mmol/L)					
Mean (SD)	3.20 (1.000)	3.26 (1.140)	3.22 (0.957)	3.16 (1.005)	1–2 1.000 2–3 1.000 1–3 1.000
SE	0.031	0.099	0.042	0.050	
95% CI	3.143–3.265	3.065–3.457	3.136–3.304	3.065–3.263	
Serum Potassium (mmol/L)					
Mean (SD)	4.60 (0.534)	4.58 (0.458)	4.60 (0.586)	4.60 (0.486)	1–2 1.000 2–3 1.000 1–3 1.000
SE	0.017	0.043	0.027	0.024	
95% CI	4.570–4.637	4.502–4.673	4.550–4.657	4.560–4.657	
Serum Sodium (mmol/L)					
Mean (SD)	141.12 (2.469)	140.73 (2.618)	141.03 (2.475)	141.36 (2.391)	1–2 0.637 2–3 0.137 1–3 0.032
SE	0.077	0.228	0.110	0.120	
95% CI	140.97–141.27	140.28–141.18	140.81–141.24	141.12–141.59	
N-terminal pro-B-type natriuretic peptide (NT-proBNP pg/mL)					
Mean (SD)	138.75 (237.297)	117.13 (164.369)	133.56 (187.244)	154.05 (310.826)	1–2 1.000 2–3 0.694 1–3 0.418
SE	7.849	14.760	8.739	17.084	
95% CI	123.35–154.16	87.89–146.33	116.39–150.74	120.45–187.66	
High-sensitivity troponin I (hs-TnI ng/L)					
Mean (SD)	6.10 (6.379)	5.41 (2.439)	6.19 (7.387)	6.23 (5.861)	1–2 0.678 2–3 1.000 1–3 0.655
SE	0.211	0.219	0.344	0.322	
95% CI	5.690–6.518	4.978–5.845	5.517–6.870	5.605–6.874	
24-h urinary potassium excretion (g/day)					
Mean (SD)	2.97 (1.128)	2.47 (1.184)	2.81 (1.031)	3.34 (1.119)	1–2 0.004 2–3 <0.001 1–3 <0.001
SE	0.034	0.101	0.045	0.055	
95% CI	2.9055–3.0419	2.2755–2.6789	2.7286–2.9070	3.2308–3.4503	
Urinary sodium-to-potassium ratio					
Mean (SD)	2.82 (1.431)	1.50 (0.727)	2.45 (0.983)	3.74 (1.531)	1–2 <0.001 2–3 <0.001 1–3 <0.001
SE	0.044	0.062	0.043	0.076	
95% CI	2.737–2.910	1.380–1.628	2.368–2.539	3.592–3.893	
Daily salt intake (g/day)					
Mean (SD)	9.32 (4.223)	3.75 (0.916)	7.41 (1.466)	13.64 (3.143)	1–2 <0.001 2–3 <0.001 1–3 <0.001
SE	0.130	0.078	0.064	0.156	
95% CI	9.0693–9.580	3.5996–3.911	7.2874–7.541	13.3389–13.955	

**Table 3:** Proportion of participants with recommended salt intake (<5 g/day) and high salt intake (>10 g/day).

	<5 g/Day (%)	>10 g/Day (%)	*p*
Gender			<0.001
Male	9.1	54.9	
Female	16.2	28.9	
Place of residence			0.0002
Rural area	9.8	45.8	
Urban area	15.5	32.6	
Adriatic region	20.8	25.6	<0.001
Continental region	7.5	49.6	
Region of residence			<0.001
Central	6.4	47.2	
Slavonia	9.1	43.1	
Northwestern	5.7	62.1	
Istria. Croatian Littoral. Gorski Kotar	14.9	32.8	
Dalmatia	19.6	24.8	
Smoking status			0.020
Never smoker	13.1	39.7	
Ex-smoker	13.7	45.0	
Smoker	17.6	29.5	
Processed meat consumption			<0.001
Never	31.0	14.8	
A few times per month	13.6	33.9	
Up to 2 times per week	11.9	45.5	
Almost every day	10.8	46.1	
Fish consumption			<0.001
Never	10.5	39.4	
A few times per month	9.0	46.2	
Up to 2 times per week	18.3	30.9	
Almost every day	30.4	39.1	
Household income, HRK * (approx. EUR **)			0.040
<2500 (<331 EUR)	8.3	41.6	
2500–3499 (332–464 EUR)	10.2	50	
3500–4999 (465–664 EUR)	12.2	44.5	
5000–10,000 (665–1326 EUR)	14.1	36.3	
>10,000 (>1327 EUR)	18.9	32.4	
Education (years)			0.040
4–8	12.3	43.0	
8–12	14.6	41.0	
>12	16.7	30.9	
Level of education			<0.001
Unskilled worker	7.7	45.5	
Highly skilled worker	9.9	41.0	
Secondary school	15.0	55.5	
Post-secondary	20.3	35.6	
University degree	25.4	24.8	
Myocardial infarction			0.005
Yes	10.5	63.1	
No	14.4	32.2	
Diabetes			0.040
Yes	5.1	41.1	
No	13.6	38.1	
Body mass index (category ***)			<0.001
1	11.1	11.1	
2	18.4	26.4	
3	14.0	36.8	
4	12.2	43.6	
5	4.4	52.9	
6	7.6	53.8	
Waist circumference (category ****)			<0.001
1	21.1	25.3	
2	13.4	38.0	
3	11.3	40.9	
Hypertension (category)			>0.05
Controlled hypertension	14.4	51.2	
Uncontrolled hypertension	12.0	43.3	
Untreated hypertension	9.3	51.2	<0.001
Normotensive individuals	17.3	33.9	

Note: *p*, *p*-value. * HRK = Croatian currency at time when study was conducted. ** EUR equivalents were calculated using the fixed conversion rate of 1 EUR = 7.53450 HRK; these amounts have not been adjusted for inflation. *** BMI category: 1 = <18.5; 2 = 18.5–24.9; 3 = 25.0–29.9; 4 = 30.0–34.9; 5= 35.0–39.9; 6 = > 40. **** Waist circumference category: 1 = men < 94 cm, women < 80 cm; 2 = men 94–101 cm, women 81–87 cm; 3 = men ≥ 102 cm, women ≥ 88 cm.

**Table 4:** Stepwise backward linear regression for salt intake, including variables statistically significant in univariate regression analysis.

	b	SE	ꞵ	*p*	95% CI
Systolic blood pressure, mmHg	0.021	0.009	0.096	0.013	0.005–0.038
Waist circumference, cm	−0.032	0.016	−0.113	0.045	−0.062–−0.001
Body surface area, m^2^	6.648	1.032	0.352	<0.001	4.622–8.675
Serum uric acid, µmol/L	−0.003	0.002	−0.054	0.182	−0.007–0.001
NT-proBNP, pg/mL	0.001	0.001	0.051	0.152	0.000–0.002
Average monthly household income, HRK *	−0.200	0.149	−0.057	0.180	−0.492–0.092
Education, years	0.789	0.316	0.132	0.013	0.168–1.410
Level of education, ordinal scale	−0.534	0.163	−0.178	0.001	−0.854–−0.214
Frequency of processed meat consumption, ordinal scale	0.705	0.176	0.145	<0.001	0.360–1.051
Frequency of poultry consumption, ordinal scale	−0.342	0.233	−0.052	0.142	−0.798–0.115
Smoking status, smoker vs. non-smoker	−0.405	0.188	−0.076	0.032	−0.774–−0.036
Region, continental vs. Adriatic, i.e., Mediterranean	1.836	0.335	0.211	<0.001	1.178–2.493

b, unstandardized regression coefficient; SE, standard error; β, standardized regression coefficient; *p*, *p*-value; CI, confidence interval. * HRK = Croatian currency at time when study was conducted.

**Table 5:** Multinomial logistic regression for high salt intake (>10 g/day).

	b	SE	*p*	OR	95% CI
Sex, male vs. female	1.21	0.277	<0.001	3.36	1.96–5.78
Smoking, non-smoker vs. smoker	0.39	0.146	0.007	1.48	1.11–1.97
Diabetes, yes vs. no	0.99	0.470	0.036	2.69	1.07–6.76
Systolic blood pressure, mmHg	0.04	0.013	0.006	1.04	1.01–1.06
Frequency of processed meat consumption, ordinal scale	0.36	0.141	0.010	1.44	1.09–1.90
Region, continental vs. Adriatic	0.44	0.090	<0.001	1.55	1.30–1.85
Lower fish consumption, ordinal scale	0.58	0.199	0.004	1.78	1.21–2.63
Average monthly household income, HRK *	−0.14	0.124	0.257	0.87	0.68–1.11
Education, years	0.05	0.203	0.787	1.06	0.71–1.57
Body mass index, kg/m^2^	0.12	0.155	0.412	1.14	0.84–1.54
Waist circumference, cm	0.24	0.195	0.216	1.27	0.87–1.87
Estimated pulse wave velocity (ePWV), m/s	0.48	0.233	0.039	1.62	1.02–2.55
Participant age, years	0.04	0.029	0.091	1.05	0.99–1.11

b, regression coefficient; SE, standard error; *p*, *p*-value; OR, odds ratio; CI, confidence interval. * HRK = Croatian currency at time when study was conducted.

## Data Availability

The original contributions presented in this study are included in the article/Supplementary Material. Further inquiries can be directed to the corresponding author.

## Supplementary Materials

The following supporting information can be downloaded at: https://www.mdpi.com/article/10.3390/life16071201/s1, Figure S1: Flow diagram of the final sample.
