# Supplementary figures and images for: Determinants of High Salt Intake in Croatian Adults: Evidence from 24-Hour Urinary Sodium Excretion in the EH-UH 2 Study
Source: Life (Basel). 2026 Jul 20;16(7):1201. doi: 10.3390/life16071201 (PMC13413395; doi:10.3390/life16071201)

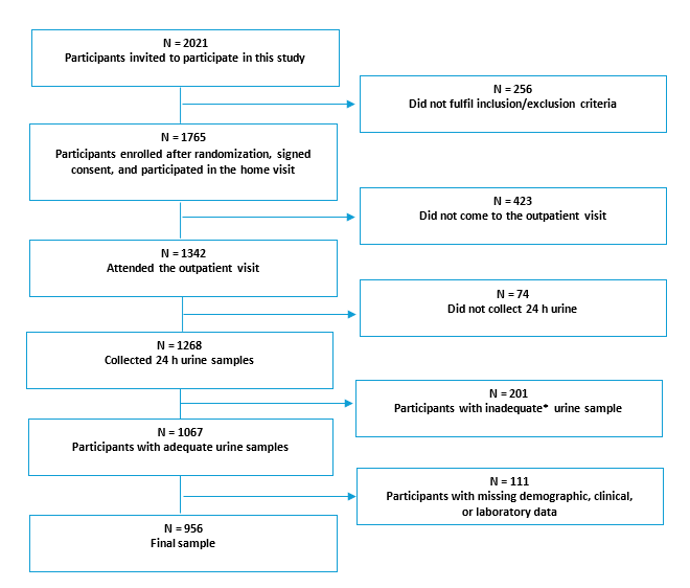

Supplement: Supplementary file 1 [file life-16-01201-s001.zip › life-4410705-supplementary Figure S1.png]
